# Minimum entropy decomposition: Unsupervised oligotyping for sensitive partitioning of high-throughput marker gene sequences

**DOI:** 10.1038/ismej.2014.195

**Published:** 2014-10-17

**Authors:** A Murat Eren, Hilary G Morrison, Pamela J Lescault, Julie Reveillaud, Joseph H Vineis, Mitchell L Sogin

**Affiliations:** 1 Josephine Bay Paul Center for Comparative Molecular Biology and Evolution, Marine Biological Laboratory, Woods Hole, MA, USA

## Abstract

Molecular microbial ecology investigations often employ large marker gene datasets, for example, ribosomal RNAs, to represent the occurrence of single-cell genomes in microbial communities. Massively parallel DNA sequencing technologies enable extensive surveys of marker gene libraries that sometimes include nearly identical sequences. Computational approaches that rely on pairwise sequence alignments for similarity assessment and *de novo* clustering with *de facto* similarity thresholds to partition high-throughput sequencing datasets constrain fine-scale resolution descriptions of microbial communities. Minimum Entropy Decomposition (MED) provides a computationally efficient means to partition marker gene datasets into ‘MED nodes', which represent homogeneous operational taxonomic units. By employing Shannon entropy, MED uses only the information-rich nucleotide positions across reads and iteratively partitions large datasets while omitting stochastic variation. When applied to analyses of microbiomes from two deep-sea cryptic sponges *Hexadella dedritifera* and *Hexadella* *cf*. *dedritifera*, MED resolved a key Gammaproteobacteria cluster into multiple MED nodes that are specific to different sponges, and revealed that these closely related sympatric sponge species maintain distinct microbial communities. MED analysis of a previously published human oral microbiome dataset also revealed that taxa separated by less than 1% sequence variation distributed to distinct niches in the oral cavity. The information theory-guided decomposition process behind the MED algorithm enables sensitive discrimination of closely related organisms in marker gene amplicon datasets without relying on extensive computational heuristics and user supervision.

## Introduction

Marker gene analyses of microbial diversity require categorizing DNA sequences into ecologically meaningful units. The two major approaches for partitioning large datasets include: (i) taxonomic classification of sequences through comparison with curated databases, for example, GreenGenes (DeSantis *et al.*, 2006; McDonald *et al.*, 2012) or SILVA (Pruesse *et al.*, 2007; Quast *et al.*, 2013) and (ii) *de novo* clustering by sequence similarity to define operational taxonomic units (OTUs). The number of unique taxonomic assignments in reference databases limits diversity descriptions because microbiologists have not defined a unified species concept (Gevers *et al.*, 2005; Doolittle and Zhaxybayeva, 2009) and molecular databases generally lack discrete name assignments for the astonishing number of uncultured microbes (Stewart, 2012). In contrast, taxonomy-independent OTU clustering divides datasets into OTUs without requiring a curated taxonomic database (Sun *et al.*, 2012). Database independence frees OTU clustering approaches from the apparent limitations of taxonomy and allows the detection of organisms that have not yet been characterized (Ley *et al.*, 2006).

FastGroup, among the first published algorithm to assign sequences to OTUs, described the three major steps employed by most contemporary OTU finding algorithms: ‘(1) compare all the sequences in a dataset to each other, (2) group similar sequences (>=97% identical to each other) together, and (3) output a representative sequence from each group' (Seguritan and Rohwer, 2001). During the last 10 years, various *de novo* approaches have facilitated OTU identification, including algorithms that typically use pairwise alignments to compute a distance matrix before grouping sequences into OTUs (DOTUR (Schloss and Handelsman, 2005), ESPRIT (Sun *et al.*, 2009), SLP (Huse *et al.*, 2010), HPC-CLUST (Matias Rodrigues and von Mering, 2014)) as well as greedy but more computationally efficient heuristics that perform sequence comparison and OTU identification simultaneously (i.e., ESPRIT-Tree (Cai and Sun, 2011), CD-HIT (Li *et al.*, 2001), UCLUST (Edgar, 2010), DySC (Zheng *et al.*, 2012)). Various software platforms, including mothur (Schloss *et al.*, 2009), QIIME (Caporaso *et al.*, 2010), CD-HIT Suite (Huang *et al.*, 2010) and VAMPS (Huse *et al.*, 2014), have adopted most of these OTU identification strategies.

Pairwise sequence identity computes the number of mismatched nucleotides between two aligned reads. However, random PCR or sequencing errors reduce the similarity between two sequences and result in false OTUs even after stringent quality filtering (Huse *et al.*, 2007; Quince *et al.*, 2009; Eren *et al.*, 2013b). To lessen the impact of sequencing errors and subsequent OTU inflation, researchers use relaxed identity thresholds for *de novo* clustering. For example, the *de facto* 97% identity often defines the diversity of clusters for 16S rRNA data. Although it reduces the number of observed OTUs, it also generates phylogenetically mixed OTUs (Koeppel and Wu, 2013). Such OTUs aggregate distinct organisms and conceal ecologically important findings (Eren *et al.*, 2013a).

Not all nucleotide positions in a dataset contribute equally towards partitioning marker gene data into ecologically meaningful units. Woese (Woese *et al.*, 1985) used short, informative oligonucleotide signatures in the 16S rRNA gene to distinguish between major bacterial clades. As recently described, oligotyping (Eren *et al.*, 2013a) also employs a form of signature analysis by using Shannon entropy (Shannon, 1948) to distinguish biologically meaningful signals from noise without requiring the calculation of pairwise sequence similarity. By relying on information-rich variable sites and discarding low-entropy nucleotide positions in a group of sequencing reads, oligotyping facilitates the identification of closely related but distinct organisms that may differ by as little as one nucleotide over the sequenced region of the 16S rRNA gene.

**Figure 1:**
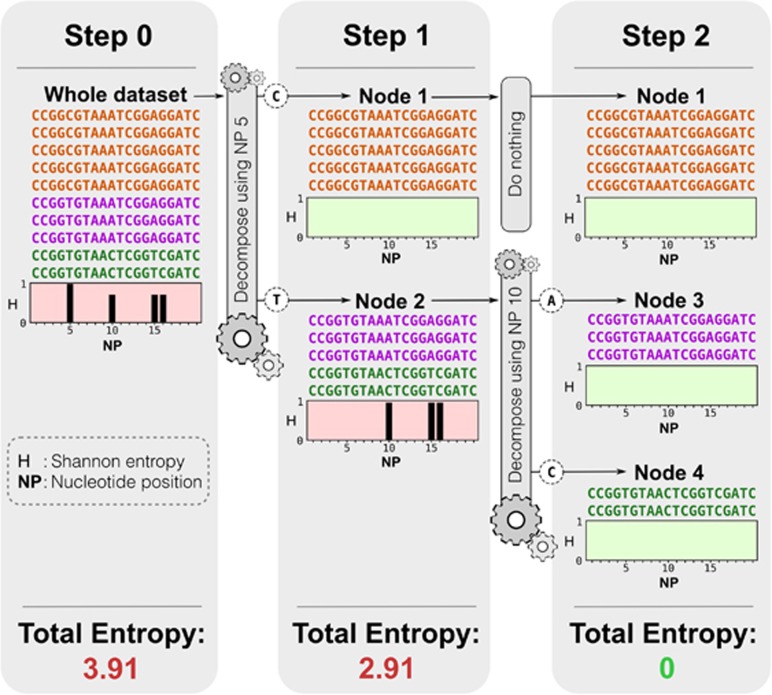
Decomposing a mock dataset of 10 reads using Shannon entropy. Each panel displays reads (identical reads have the same color) within individual nodes, the corresponding entropy profiles (indicated by black bars at each position) and the total entropy value for all reads in the node. Between steps, any node that has a Shannon entropy value greater than 0 is decomposed using only the nucleotide position corresponding the highest or left-most entropy position, assuming the left-most side of the read represents the higher quality end. When a node is decomposed, entropy is reanalyzed for the newly generated nodes. In this mock example, two decomposition steps identified final units with each step using one entropy location.

Oligotyping different high-throughput sequencing datasets has resolved unexplained diversity within taxa and OTUs (Eren *et al.*, 2011; Mclellan *et al.*, 2013; Apprill *et al.*, 2014; Eren *et al.*, 2014; Maignien *et al.*, 2014; Reveillaud *et al.*, 2014). However, oligotyping has characteristics that sometimes limit its applicability to environmental datasets. First, oligotyping performs optimally when applied to closely related taxa as the great number of high-entropy locations among distantly related organisms renders the supervision step arduous (Eren *et al.*, 2013a). Second, oligotyping requires a preliminary OTU clustering or taxonomic classification to identify closely related taxa suitable for analysis. Oligotyping is not a stand-alone approach and its improvements cannot be applied directly to the entirety of a sequencing dataset. Nevertheless, oligotyping shows the potential of entropy to partition mixed information into homogenous units by using a fraction of available sequencing data, and without pairwise sequence alignment and comparison (Figure 1).

**Figure 2:**
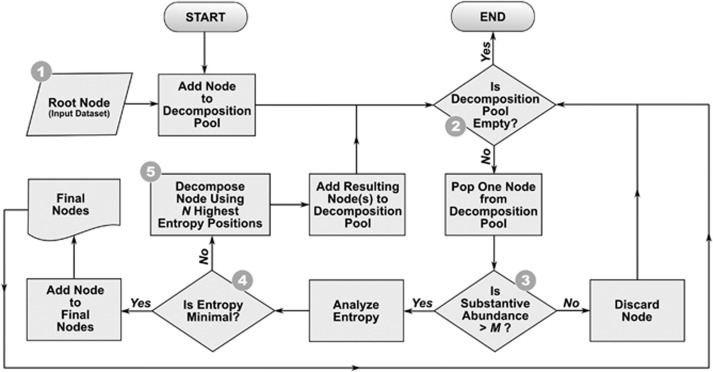
Flowchart of the MED loop. The input dataset is decomposed into internal MED nodes using high-entropy positions. An internal node is converged if no discernible entropy peak is left for further decomposition and is called a final or terminal MED node.

We have developed and employed a new algorithm, ‘Minimum Entropy Decomposition' (MED), to partition high-throughput sequencing datasets into ecologically meaningful and phylogenetically homogeneous units by extending the underlying principles of oligotyping to entire marker gene datasets. MED uses information uncertainty among sequence reads to iteratively decompose a dataset until the maximum entropy criterion is satisfied for each final unit (i.e., until there is no entropy left to explain; Figure 2). In contrast to oligotyping, MED requires no user supervision, no preliminary classification or clustering result, and can be applied to the entire dataset instead of only a group of closely related taxa.

We used two datasets to demonstrate the utility of MED: a new V4-V5 rRNA gene dataset from previously described host microbiomes of deep-sea sponges (Reveillaud *et al.*, 2014) and a publicly available human oral microbiome dataset (The Human Microbiome Project Consortium, 2012b). We compared MED analysis results to taxonomic analysis and *de novo* OTU clustering using QIIME with UCLUST, which among several methods, has often been used in studies of the Human Microbiome Project (The Human Microbiome Project Consortium, 2012a) and the Earth Microbiome Project (Gilbert *et al.*, 2010).

## Materials and methods

### Minimum Entropy Decomposition

The algorithm iteratively partitions a dataset of amplicon sequences into homogenous OTUs (‘MED nodes') that serve as input to alpha- and beta-diversity analyses. MED inherits the core principle of oligotyping (Eren *et al.*, 2013a) and uses Shannon entropy to identify information-rich positions within an internal node. Entropy increases proportionally to the amount of variability in a nucleotide position and MED uses high entropy positions to decompose a node into child nodes. A nucleotide position that directs a decomposition step will have zero entropy in child nodes. Hence, the increasing number of identified nodes decreases the cumulative entropy in the dataset (Figure 1). For each cycle of decomposition, an entropy profile that does not contain any discernible entropy peak defines the stop condition of the algorithm for a given node. If a node's entropy profile is not uniform, indicating that it has not *converged*, MED will decompose the node further. Figure 2 gives an overview of the algorithm.

The MED algorithm operates on high-throughput sequencing datasets without requiring an initial DNA sequence alignment step. Like oligotyping, MED requires length differences among sequenced reads to represent biologically meaningful variation rather than indels derived from systematic sequencing errors such as homopolymer indel events commonly encountered with 454/Roche technology. If read length differences represent biologically meaningful variation, appending gap characters to the short reads would be sufficient to finalize the data; otherwise, an alignment step would be necessary. A terminal MED node corresponds to a high-resolution OTU identified through a decomposition process that resembles a bifurcating tree, rather than through a *de novo* clustering strategy. The algorithm can detect biologically meaningful differences between closely or distantly related sequences in large datasets without requiring CPU-intensive alignment. Although subsampling is not mandatory for MED, it must be considered if the number of reads across samples in a dataset differ by multiple orders of magnitude to avoid biases in entropy results. The MED pipeline assumes that the input dataset is quality-filtered. Most widely used quality-filtering approaches trim low-quality ends of sequencing reads (Minoche *et al.,* 2011; Bokulich *et al.,* 2013); to eliminate artificial read length variation for MED analysis, reads that require trimming should be discarded prior to analysis.

MED (see Figure 2) initially adds the input dataset (Step 1) to the decomposition pool as a node for analysis. The ‘decomposition pool' represents a transient collection of nodes that have not yet *converged*. An MED node (Step 4) has *converged* if it has a flat entropy profile. The algorithm iteratively evaluates entropy for internal nodes until exhaustion of the decomposition pool (Step 2). At each iteration, MED removes a single node from the pool for analysis. The removed node is either discarded, identified and stored as a final MED node, or further decomposed into child nodes that are added to the decomposition pool. MED discards nodes that do not satisfy the minimum substantive abundance (*M*) criterion (Step 3). It uses *M* to filter noise as described for oligotyping (Eren *et al.*, 2013a): if the most abundant unique sequence of a node is smaller than the user-defined value of *M*, MED will remove it from the analysis. We recommend setting *M* to *N*/10 000 or larger, where *N* equals the number of reads in the dataset. A node that satisfies *M* is subjected to Shannon entropy analysis to identify information-rich positions (Step 4), and the node is identified as a terminal node if it has a flat entropy profile. Owing to sequencing errors, entropy is rarely zero for a given nucleotide position in large nodes, hence MED uses a threshold (*m*), below which entropy is treated as zero. The *m* parameter is determined dynamically during the runtime for each node, and by default it decreases from 0.0965 (the expected entropy generated by a sequencer with 1% error rate) proportional to the ratio of the size of a node and the frequency of the most abundant unique read in the dataset. The following equation demonstrates the default *m* heuristics in the MED algorithm developed empirically based on our experience with Illumina HiSeq and MiSeq datasets,


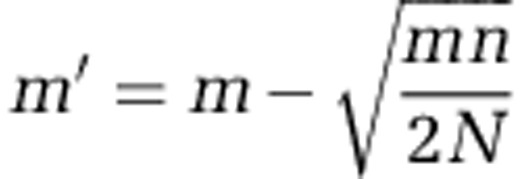


where *m'* is the normalized *m* for a node with *n* reads found in a dataset where the frequency of the most abundant unique read is *N*. If the entropy profile of a node is not minimal, that is, there exists one or more entropy peaks greater than the normalized *m*, MED proceeds to decompose the node (Step 5). The parameter *c* defines the maximum number of nucleotide positions with entropy values greater than *m* for decomposing every node throughout the MED process. A small *c* requires additional iterations to reach convergence, whereas a large *c* would discard more reads according to the *M* criterion. In our implementation of MED, we determined 4 to be a reasonable default for *c*; however, the user can select a different value. When an intermediate node is decomposed (Step 5), the resulting child nodes join the decomposition pool for subsequent iterations of MED analysis. This loop continues until all nodes are analyzed and the decomposition pool is empty. Along with basic visualizations and reports, a completed MED run will generate standard output files (e.g., observation matrices, FASTA files for representative sequences and network descriptions). Source code and user manuals for MED can be found at http://oligotyping.org.

### Sample collection and handling

#### Sponge data

We sequenced the V4-V5 region of bacterial rRNA genes from 19 deep-sea sponge and 5 control water samples. Samples were collected at locations along >5000 km of the European margins and spanned wide bathymetric gradients (130–958 m) (Reveillaud *et al.*, 2014).

#### Human Microbiome Project data

The Human Microbiome Project Consortium (2012a) describes the sample collection and pyrosequencing of the oral microbiome dataset. Of the 242 individuals who participated in the study, we included only individuals who were sampled at each oral site (*n*=148 for V3-V5 data) (Eren *et al.*, 2014).

### Library preparation and sequencing

We constructed amplicon libraries from sponge samples that span the V4-V5 16S rRNA region (Supplementary Methods). Supplementary Table S1 describes the 16S-specific primers and the sequencing adaptors for paired-end sequencing on the Illumina MiSeq platform (Illumina Inc., San Diego, CA, USA) using 2 × 250 cycles. V3-V5 pyrosequencing reads (250 nt in length) from a publicly available oral microbiome study (The Human Microbiome Project Consortium, 2012b) represent samples from nine sites in the human mouth and pharynx (subgingival plaque, supragingival plaque, buccal mucosa, keratinized gingiva, tongue dorsum, hard palate, saliva, palatine tonsils and throat).

### Quality filtering

We merged and quality-filtered the partially overlapping V4-V5 paired-end reads of the sponge microbiome dataset using our Illumina Utilities library (https://github.com/meren/illumina-utils; see Supplementary Methods). To minimize the impact of read count variation, we randomly subsampled the sponge dataset to a maximum of 20 000 reads per sample. After quality filtering and subsampling, the sponge dataset represented 373 474 reads from 24 samples. Supplementary Table S2 reports the number of raw and quality-filtered reads per sample. VAMPS (Huse *et al.*, 2014) posts the filtered sponge datasets under project ID MER_MED_Bv4v5 and the NCBI Sequence Read Archive hosts the unprocessed sequences under accession SRP042371. The Human Microbiome Project oral microbiome dataset from 1332 samples (9 oral sites sampled from 148 individuals) initially contained >7 M quality-filtered reads. The Human Microbiome Project Consortium's quality filtering method removed reads with one or more ambiguous base calls and those with a homopolymer of 8 nt or longer. Reads trimmed from the 3′ end used a sliding window of average quality score (The Human Microbiome Project Consortium, 2012a). We re-trimmed the data to a consistent length of ∼235 nt and discarded short reads (Eren *et al.*, 2014), resulting in 5  926 860 quality-filtered reads.

### Taxonomic classification, OTU clustering and MED analyses

We used GAST (Huse *et al.*, 2008) to assign taxonomy to our reads individually. For OTU clustering, we used QIIME v1.5 (Caporaso *et al.*, 2010) with UCLUST (Edgar, 2010) in *de novo* mode *via* the *pick_otus.py* script. We ran UCLUST with default parameters and generated clusters with 97% similarity threshold. We used the open-source MED pipeline version 1.2 for MED analysis. To filter noise, we set the minimum substantive abundance criterion (*M*) to 100. We removed GAST taxa and OTUs that were represented by fewer than 100 reads for comparability to the MED results. To assess the performance of MED with respect to UCLUST, we subsampled the oral microbiome dataset incrementally and analyzed each file separately with both methods. We used the default parameters for both, except for the addition of the —quick flag for MED to time the raw decomposition. ‘Unit' refers to the end product of a partitioning method: a GAST taxon, an OTU, an oligotype or an MED node. The most abundant unique sequence will represent each unit. Supplementary Table S2 and S3 report observation matrices and representative sequences for each method.

### Statistical analyses and visualization

We evaluated sample group variances in the sponge microbiome and human oral microbiome datasets explained by each method using the betadisper function in the vegan package (Anderson, 2006) for R (R Core Team, 2013) and reduced distances produced from Horn dissimilarity coefficients to principal coordinates, which embeds sample coordinates in Euclidean space. Non-parametric multivariate analysis of variance on the dataset coordinates (using the ADONIS function in the vegan package identified the ratio of between-group variance to within-group variance and the proportion of total variation associated with different sites in the human oral cavity. To investigate the recovery of previously identified taxa, we searched the representative sequences of taxa, OTUs and MED nodes in the oral microbiome dataset against the Human Oral Microbiome Database (HOMD) (Chen *et al.*, 2010) version 12.0 (obtained from http://www.homd.org on 23 April 2014), using blastn (Altschul *et al.*, 1997) v2.2.26+. We performed network analyses using Gephi (Bastian *et al.*, 2009) with ForceAtlas2 layout (http://gephi.org). We used http://raw.densitydesign.org/ to visualize alluvial diagrams.

## Results

### MED resolves microbiome differences between two closely related deep-sea sponge species

**Figure 3:**
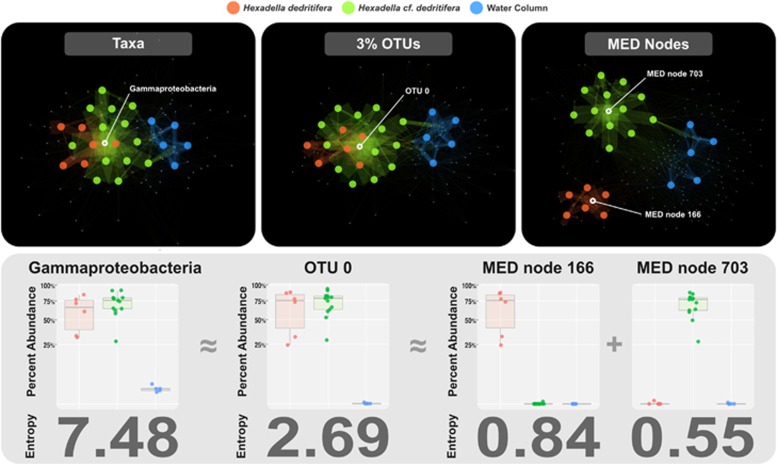
Network analysis of *Hexadella* spp. and water column samples with respect to identified taxa, 3% OTUs and MED nodes. The bottom panel shows the percent abundance of taxon Gammaproteobacteria, OTU #0 and two MED nodes among sample groups and the total entropy contained in each unit. Only the MED analysis distinguished between the overlapping microbiomes of closely related *Hexadella* species.

The deep-sea sponge cryptic species *Hexadella dedritifera* and *H.* cf. *dedritifera* are distinguishable only through genetic surveys (Reveillaud *et al.*, 2010). We analyzed the microbiomes of six *H. dedritifera*, 13 *H. cf. dedritifera* and 5 local water samples. Analysis of 373 474 quality-filtered reads with GAST, OTU clustering and MED detected 80 taxa, 91 OTUs and 187 MED nodes, respectively. GAST mapped nearly 50% (186 562 sequences) of the reads to the class Gammaproteobacteria, which accounts for 84.5% and 91.3% of the reads in *H. dedritifera* and *H.* cf. *dedritifera,* respectively (see Figure 3). UCLUST identified one dominant cluster, OTU #0, containing 96.2% of all reads that GAST mapped to the Gammaproteobacteria class-level. The relative abundance of OTU #0 ranged from 28.5 to 93.3% (average 71.7%) in *H.* cf. *dedritifera*, and ranged from 24.3 to 86.5% (average 63.5%) in *H. dedritifera*. OTU #0, the most abundant OTU in both cryptic species represented most of the overlap between the *Hexadella* spp. bacterial communities. Thus OTU analysis showed little improvement over taxonomic analysis (Figure 3). However, MED split OTU #0 into two terminal nodes that showed strong differential distribution between the two *Hexadella* species. MED Node 703 significantly associated with *H.* cf. *dedritifera,* whereas MED Node 166 significantly associated with *H. dedritifera*. Representative sequences of these two MED nodes showed only 3 nt differences, or 99.2% sequence identity.

### Improved partitioning of oral microbiome sites by MED and oligotyping

**Figure 4:**
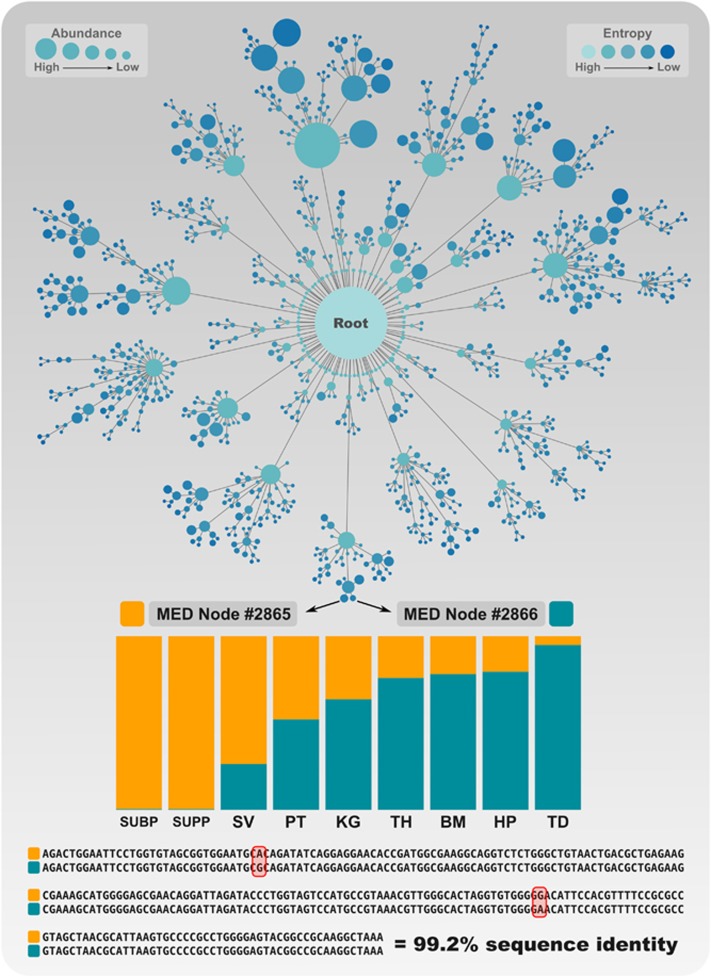
The topology of the MED process. The top panel shows the decomposition of the oral microbiome dataset composed of ∼6 M reads into intermediate and final nodes by MED. Two final nodes in the topology are marked (nodes #2865 and #2866) and the bar-chart plot shows the their distribution across oral sites in human mouth; subgingival plaque (SUBP), supragingival plaque (SUPP), saliva (SV), palatine tonsils (PT), keratinized gingiva (KG), throat (TH), buccal mucosa (BM), hard palate (HP) and tongue dorsum (TD). The lower panel shows the alignment for the representative sequences of the two nodes, which are 99.2% identical.

**Figure 5:**
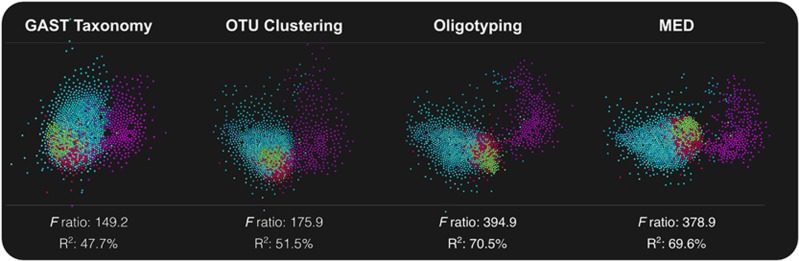
Comparison of oral microbiota sample distribution with network analysis and multivariate analysis of variance results. The top panel shows network analysis results for each method. In each plot, dots represent oral samples from the nine oral sites. For visual clarity, we colored plaque samples with purple, buccal mucosa with green, keratinized gingiva samples with red, and the remaining samples from throat, tonsils, tongue dorsum, hard palate and saliva with cyan. The bottom panel shows the ratio of between-group variance/within-group variance (F-ratio) and the proportion of total variation captured by the different oral sites defined in the model (R^2^) for GAST taxonomy, OTU clustering, oligotyping and MED.

We re-analyzed an oral microbiome dataset of ∼6 million V3-V5 rRNA sequences (The Human Microbiome Project Consortium, 2012a) using taxa, *de novo* OTUs and MED nodes. The previous report of 481 oligotypes (Eren *et al.*, 2014) compares to GAST and clustering detection of 122 taxa and 329 OTUs, respectively. Using the same dataset, MED identified 858 terminal nodes. UCLUST analyzed the oral microbiome dataset two times faster than MED, which took ∼25 min in our simulation. Runtimes for both algorithms increased linearly with increasing number of reads (Supplementary Figure S1). Figure 4 demonstrates the tree-like topology of the decomposition process with intermediate and terminal nodes identified in the oral microbiome dataset. Multivariate analysis of variance compared the captured proportion of variation in this dataset associated with the oral sites using all four methods. Multivariate analysis of variance analysis of the same dataset produced two comparison metrics: *F*-ratio and R^2^. Increasing similarity of samples collected from the same oral site compared to samples collected from other sites increases the *F*-ratio. The R^2^ value indicates the proportion of total variation captured by the model. Oligotyping and MED partitioned the dataset with increased resolution relative to taxon-based and OTU-based analyses (Figure 5).

### MED recovers more organisms from the HOMD

The curated HOMD holds 688 near-full-length rRNA gene reference sequences for microbial taxa isolated from healthy and diseased human oral cavities (Dewhirst *et al.*, 2010). We evaluated the ability of the four analytical methods to identify known oral microbes. We compared each sequence in the oral microbiome dataset with the HOMD and recovered 516 matches with 100% identity and coverage. Of these, 248 occurred more than 100 times in our dataset. This defines the maximum number of HOMD taxa that we can identify in the oral microbiome dataset that contain at least 100 members in a taxonomic group, cluster or node. The remaining perfect hits to the HOMD that occurred fewer than 100 times represented 0.056% of the oral microbiome dataset. BLAST queries using representative sequences of each taxon, OTU and MED node identified 67, 112 and 235 matches, respectively, to sequences in the HOMD database using the criteria of 100% identify and coverage. When we limited our comparison to only the 329 most abundant MED nodes, numerically equal to the 329 OTUs found, the MED nodes still matched more HOMD taxa (138) than did the OTUs (112). Representative sequences of taxa, OTUs and MED nodes, respectively, identified 27%, 45% and 95% of the strains described in HOMD using sensitivity settings of 100 or more occurrences. By partitioning a dataset of ∼6 M reads into 858 terminal nodes, MED recovered 95% of what the unique reads would have recovered at the same sensitivity.

### MED nodes are ecologically relevant

**Figure 6:**
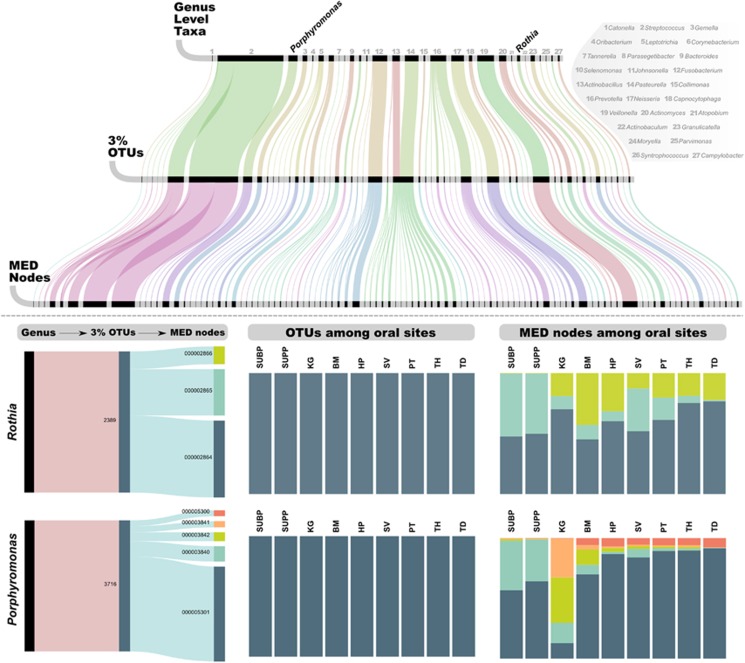
Alluvial diagram of the relationship between the top 100 MED nodes, OTUs and taxa in the oral microbiome dataset. Three horizontal lines identify the units for a given level. The size of each black bar represents the abundance of that unit in the dataset. The total number of reads represented in each horizontal bar is identical and makes up 83% of the oral microbiome dataset. Vertical lines demonstrate how these reads are grouped differently by each method. The two examples in the bottom panel demonstrate the distribution of OTUs and MED nodes associated with two taxa, *Rothia* and *Porphyromonas* across oral sites; subgingival plaque (SUBP), supragingival plaque (SUPP), keratinized gingiva (KG), buccal mucosa (BM), hard palate (HP), saliva (SV), palatine tonsils (PT), throat (TH) and tongue dorsum (TD).

The larger number of exact sequence matches to the HOMD reference database for MED nodes (235 matches) relative to OTUs (112 matches) from the oral microbiome dataset reflects MED's ability to partition the data into more phylogenetically homogeneous units. To investigate the impact of an increased number of units on ecological inference, we explored the source OTUs and taxa for reads that make up the most abundant 100 MED nodes using an alluvial diagram (Figure 6). A total of 4 936 104 reads (83% of the entire dataset) accounted for the 100 most abundant MED nodes. For clarity, we did not draw connections that corresponded to fewer than 1000 reads. This analysis accounted for 29 taxonomic groups and 57 OTUs. In most cases, individual taxa split into multiple OTUs, which then split into multiple MED nodes. However, we observed at least one example where the genera *Actinobacillus* and *Pasteurella* (taxa #13 and #14 in Figure 6) converged into one OTU. Sometimes all three methods agreed; for instance, reads that resolved to either *Catonella* or *Oribacterium* (Taxon #1 and #4 in Figure 6) also resolved to one OTU, and one MED node. We further investigated reads that resolved to *Rothia* and *Porphyromonas* by considering their ecological context. All *Porphyromonas* and all *Rothia* reads mapped to single OTUs, yet MED split each of these OTUs into multiple terminal nodes (3 and 5 nodes, respectively). The distribution of MED nodes in both cases displayed differential distribution among oral sites (Figure 6). For example, MED node #2866 identified a group of *Rothia* that did not occur in plaque. However, MED node #2865 represents >50% of the *Rothia* taxa in plaque, but lower fractions of the *Rothia* populations from other oral sites. Despite their differential site distribution, MED nodes #2866 and #2865 (Figure 4) differ by only two nucleotides (99.2% sequence identity). Similarly, MED nodes classified to *Porphyromonas* show distinct distribution patterns of very closely related taxa that did not resolve by OTU clustering or taxonomic analysis. Supplementary Figure S2 provides additional examples of meaningful MED node distribution patterns for *Streptococcus*, *Fusobacterium*, *Neisseria* and *Bacteroides*.

## Discussion

MED partitions large marker gene datasets into terminal nodes that can share higher levels of sequence similarity than OTU clusters. MED identified organisms in two example datasets that differ by only a few nucleotides, yet distribute differently across environments, and recapitulated published oligotyping results. Instead of requiring pairwise sequence alignment or taxon assignments, MED uses a fraction of the available nucleotide variation in an iterative process that relies upon the most information-rich sites to decompose large datasets.

In our sponge microbiome analysis, MED detected substantial differences in microbial communities between two closely related deep-sea sponge species, *H.* *cf.* *dedritifera* and *H. dedritifera* that other approaches missed. Cluster analysis identified the highly abundant OTU #0, which included a large number of reads that GAST mapped only to the class Gammaproteobacteria. MED analysis of the dataset split OTU #0 into two distinct nodes with representative sequences that showed only 3 nt differences (99.2% sequence identity). Although *H.* *cf*. *dedritifera* and *H. dedritifera* can occur sympatrically (i.e., on the Irish continental margin), MED revealed highly specific sponge–Proteobacteria associations at a very fine taxonomic scale. Gammaproteobacteria reads that cluster together in OTU #0 represent abundant and ecologically distinct organisms that might serve different functions for the sponge host or out-compete other variants in response to a given host environment. Species-specificity sometimes reflects complex genetic interdependence between a host and its associated bacteria (Bulgheresi *et al.*, 2006; Gourdine *et al.*, 2007; Mandel *et al.*, 2009; Franzenburg *et al.*, 2013).

Unlike oligotyping, MED identifies subtle nucleotide variation among high-throughput sequencing reads without user supervision. We recently used oligotyping to explore the diversity of very closely related commensal and pathogenic organisms within distinct phyla that represent more than 99% of the sequencing data in a human oral microbiome dataset (Eren *et al.*, 2014). Our supervised analysis of each major phylum revealed that some organisms differing by as few as a single nucleotide showed dramatically different distributions among oral sites and among individuals (Eren *et al.*, 2014). Here we re-analyzed the same dataset to compare oligotyping with taxonomy, OTU clustering and MED results. Our analyses showed strong congruence between MED and oligotyping compared with taxa and OTUs. MED nodes captured a larger proportion of variation associated with different oral sites and better described the distribution of very closely related organisms that occupied different niches in the human oral cavity.

Proper sequence partitioning will reduce the number of observed units in a given dataset without sacrificing ecological inference. In the absence of PCR or sequencing errors, unique sequences in a dataset would serve as ideal proxies for genomic signatures in microbial communities. However, even the most stringent quality-filtering methods applied to large marker gene datasets will retain many more unique sequences than the true number of different organisms. Hence the dilemma: partitioning strategies must account for subtle differences between highly similar sequences to accurately represent every organism, while not inflating the number of observed groups artificially. A thorough comparison of partitioning methods that strive to achieve this goal requires determining the minimum number of units in a dataset that accurately represent every organism, which *de novo* investigations cannot determine. However, a curated database can serve as a reference to demonstrate how many well-described organisms each method recovers. In our analysis of the oral microbiome, we used the phylogenetically curated HOMD of 688 near full-length 16S rRNA gene sequences from mostly cultivated organisms as a reference. Among the ∼230 000 unique sequences in our oral microbiome dataset, only 248 unique sequences with a frequency of 100 or higher matched an entry in HOMD. Hence, the goal for any partitioning method is to generate units with representative sequences that match 248 sequences in HOMD without inflating the total number of units. All three unsupervised methods reduced the number of observed units by orders of magnitude compared to the number of unique sequences; however, MED representative sequences recovered 95% of expected HOMD entries, whereas the representative sequences of taxa and OTUs recovered only 27% and 45%, respectively. MED's ability to appropriately identify more organisms from the oral microbiome predicts similar improvements in analyses of other environments.

Computational heuristics for *de novo* OTU clustering continue to improve; however, the core concept of defining cluster membership according to measures of sequence similarity (e.g., evolutionary distances, k-tuple distances, etc.) neglects the theoretical advantages of considering only information-rich sites in long sequence reads. The number of nucleotide positions that can differ between two reads in an OTU (i.e., the heterogeneity within the OTU), increases linearly with read length. Two 100 nt sequences or two 300 nt sequences with 97% identity can cluster together, but the longer sequence will contain on average three times as many information-rich sites, each of which might further resolve the cluster into additional homogeneous units. One solution is to increase the similarity threshold, as previously proposed (Stackebrandt and Ebers, 2006); however, this approach does not scale. Although longer reads provide increased phylogenetic resolution, they will likely possess larger numbers of sequencing errors, precluding the use of higher similarity thresholds. Here, even a very conservative similarity threshold of 99%, while greatly increasing the number of observed OTUs, would have failed to identify distinct microbiomes of closely related sponge species or distinguish ecologically distinct members of the oral microbiome that share more than 99% sequence identity (Figure 6). The sensitivity of computational methods that partition sequencing datasets should be agnostic to read length and pairwise sequence similarity.

Our MED results demonstrate the advantage of relying on information theory rather than pairwise sequence similarity to sensitively define ecologically relevant units in a dataset. It differs in important ways from commonly used clustering approaches. MED iteratively partitions sequencing datasets of marker genes into phylogenetically homogeneous units using entropy, but imposes minimal computational heuristics by disregarding sequence similarity or phylogenetic relationships between reads. As MED does not perform pairwise sequence alignment and similarity assessment during decomposition, it utilizes a fraction of the available nucleotide data in a dataset. Instead of grouping reads based on sequence similarity, it splits groups of reads into more refined units incrementally based on the nucleotide positions that present dissimilarities identified by the entropy analysis. These differences allow MED to detect and explain subtle nucleotide variation more effectively and identify distinct organisms that are as few as one nucleotide apart regardless of the length of the sequenced region.

No currently available method addresses every potential artifact associated with large marker gene datasets. Sequencing errors, PCR errors, alignment artifacts and failure to resolve closely related taxa lead to inaccurate assessments of microbial community diversity and ecology. Analytical methods that mitigate the influence of sequencing errors through *de novo* clustering at a predefined level of sequence similarity generally sacrifice sensitivity as they fail to resolve very closely related taxa that track ecological context. In contrast, MED provides very a high-resolution depiction of microbial communities while recapitulating the oligotyping results without user supervision. To eliminate ‘noise' or false nodes caused by sequencing error, it employs the criterion ‘minimum substantive abundance' (*M*) to dictate the removal of any intermediate or terminal MED nodes with a representative sequence that occurs in the dataset less than *M* times.

One notable feature of MED from a computational perspective is that individual nodes are analyzed independently from each other throughout the decomposition process (Figure 4). Thus the algorithm eliminates the need for shared memory space for subsequent steps of analysis, which makes significant performance gains possible through distributed and parallel implementations. MED is a *de novo* approach; therefore MED nodes, in theory, are not comparable across studies. However, as a fully resolved MED node will have minimal phylogenetic mixture, to what extent the representative sequences of MED nodes can be compared across studies warrants further investigation.

In this study, we demonstrated the capacity of MED, a sensitive approach that explains the diversity of closely related organisms in high-throughput marker gene datasets regardless of read length and percent similarity. MED can facilitate the identification of keystone organisms, representative sequences of which can be used as biomarkers or guide in-depth, hypothesis-driven genomic analyses.
