# Supplementary figures and images for: Minimum entropy decomposition: Unsupervised oligotyping for sensitive partitioning of high-throughput marker gene sequences
Source: ISME J. 2014 Oct 17;9(4):968–79. doi: 10.1038/ismej.2014.195 (PMC4817710; doi:10.1038/ismej.2014.195)

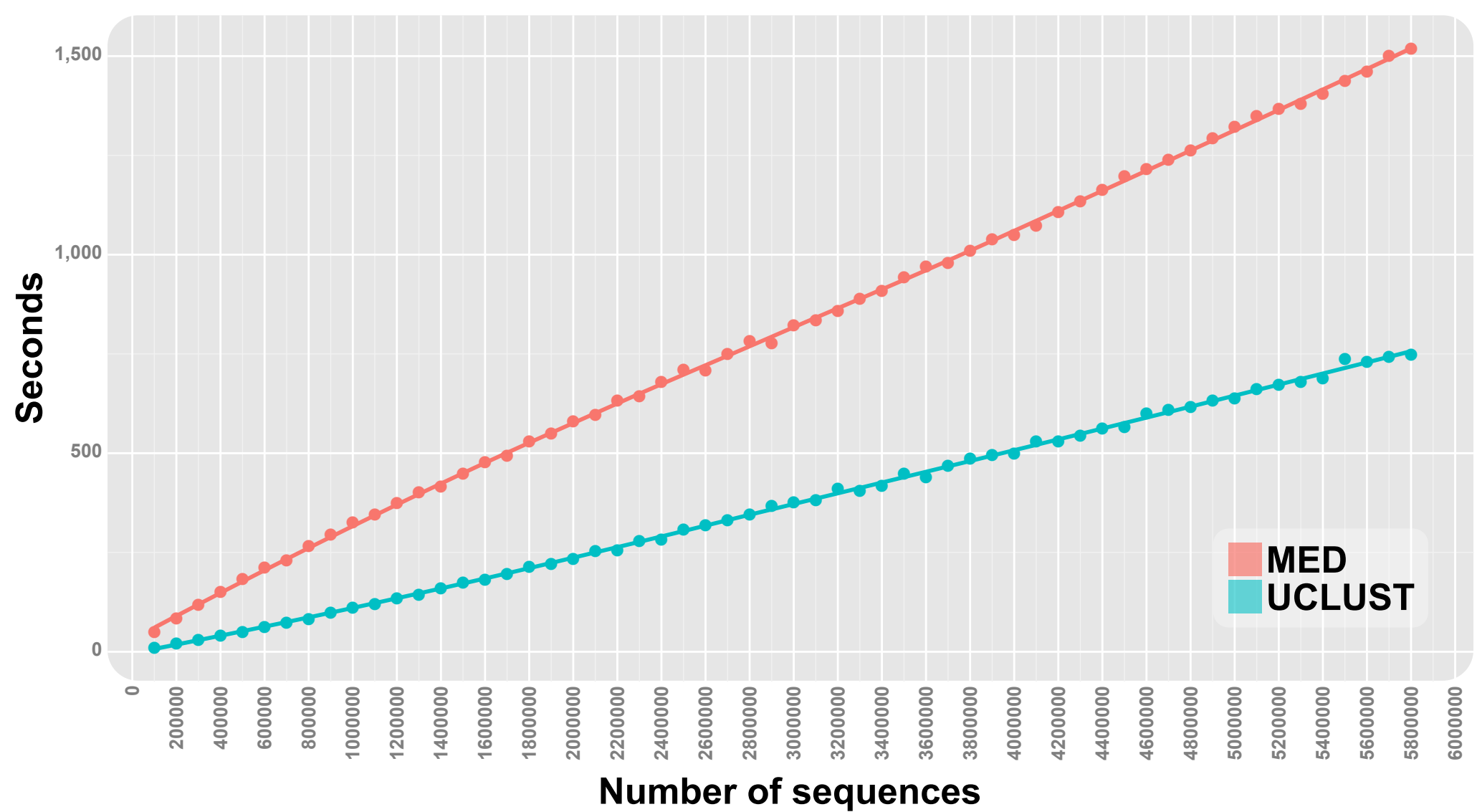

Supplement: Supplementary Figure 1 [file ismej2014195x1.pdf]

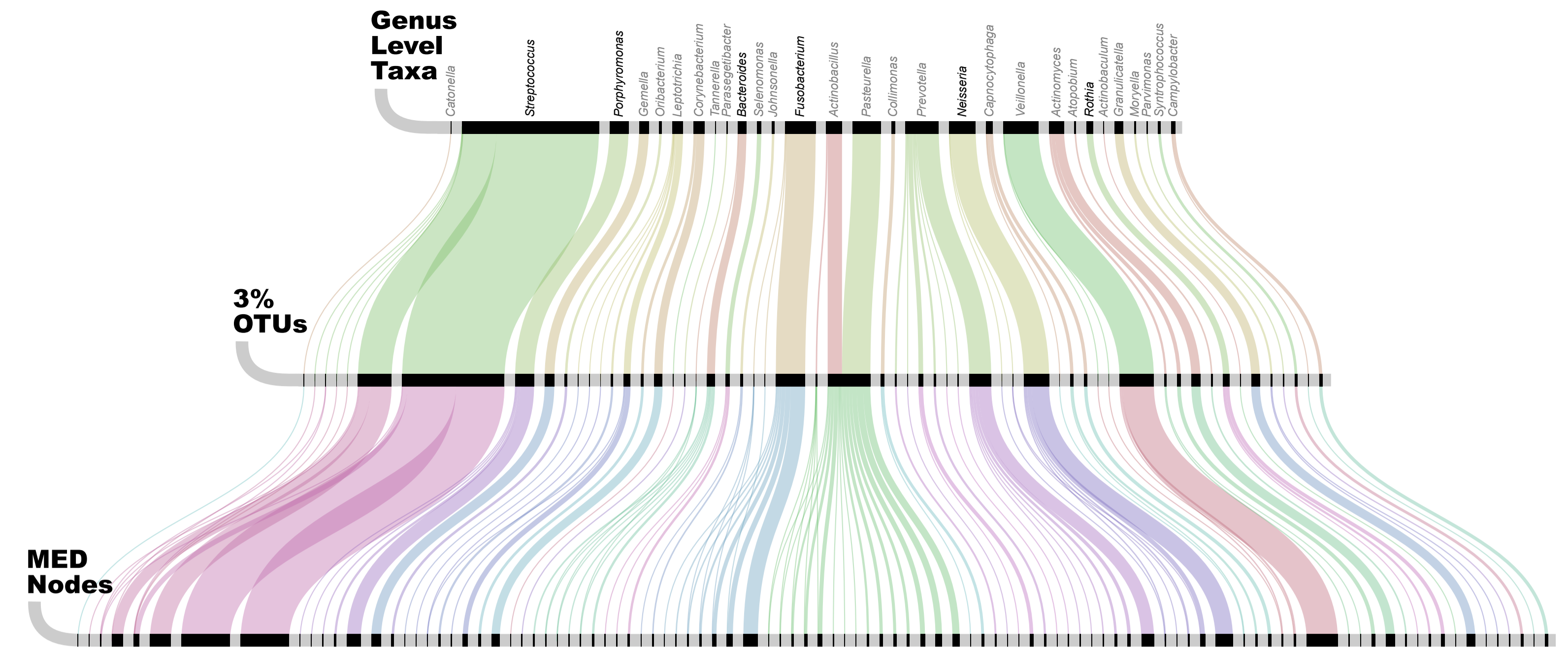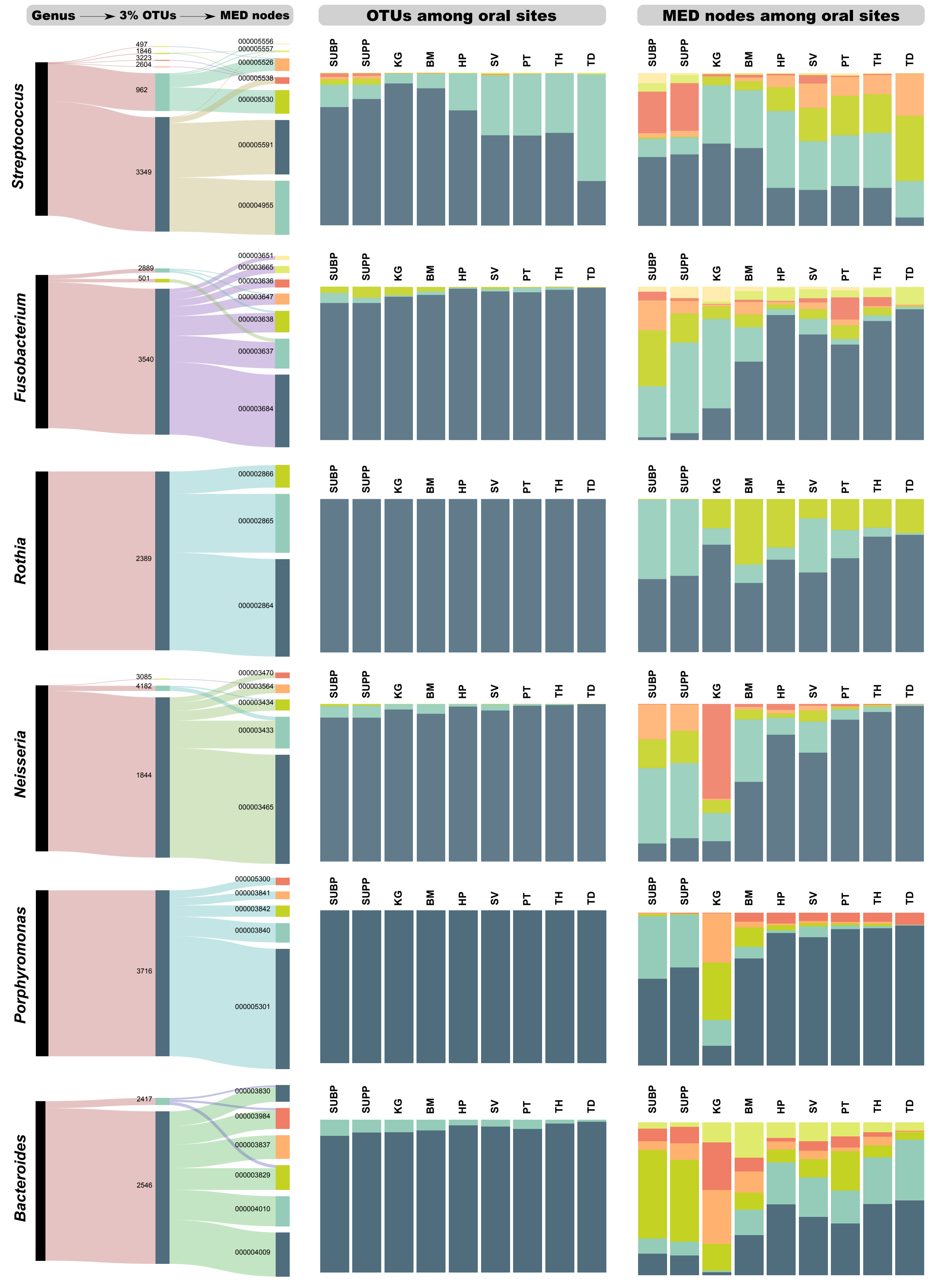

Supplement: Supplementary Figure 2 [file ismej2014195x2.pdf]
