# Supplementary material for: Minimum entropy decomposition: Unsupervised oligotyping for sensitive partitioning of high-throughput marker gene sequences
Source: ISME J. 2014 Oct 17;9(4):968–79. doi: 10.1038/ismej.2014.195 (PMC4817710; doi:10.1038/ismej.2014.195)
Supplement: Supplementary Information [file ismej2014195x3.pdf]

Supplementary Methods

Library preparation and sequencing. Amplicon libraries from sponge samples span the V4-V5 16S rRNA region (paired-end sequenced between 518F and 926R). The 16S-specific primers and the sequencing adaptors are shown in Supplemental Table X. The primers contain the bridge adaptors necessary for clustering, sequencing primer binding sites, forward or reverse 16S-specific primer, and an in-line multiplex identifier (MID; forward primer) or index that is sequenced in a separate indexing read (reverse primer). We use a combination of in-line MID and index to multiplex samples. The 16S-specific primers contain degenerate sites or, in the case of 926R, represent a combination of three distinct oligonucleotides in order to capture broad eubacterial diversity. The V4-V5 amplicons were generated using a reaction mix containing 1X Platinum HiFi Taq polymerase buffer, 2 mM $MgSO_4$, 0.2 mM dNTPs, 0.4 uM of the forward and reverse primers, 10-30 ng genomic DNA, and 10 units of Platinum HiFi Taq polymerase (Life Technologies, Carlsbad CA) in a volume of 100 µl. This mix was divided into three replicate reactions before cycling. Cycling conditions were: an initial 94°C, 3 minute denaturation step; 30 cycles of 94°C for 30 s, 60°C for 45 s, and 72°C for 60 s; and a final 2 minute extension at 72°C. The triplicate PCR reactions were pooled after amplification and purified to exclude primer-dimers using Ampure XP (Beckman Coulter, Indianapolis IN) in a 1:1 volume ratio according to the manufacturer's protocol and eluted in 20 µl of Qiagen buffer EB (Qiagen, Valencia CA). Libraries were quantitated using PicoGreen QuantIt (Life Technologies, Carlsbad CA) and pooled in equimolar amounts. The final pool was quantitated using the KAPA library quantification protocol (Kapa Biosystems, Boston MA). Libraries were sequenced on an Illumina MiSeq 250 cycle paired-end run. The combination of CASAVA 1.8.2 to identify reads by index and a custom Python script that resolved barcodes demultiplexed the datasets.

Quality filtering. We used out our Illumina Utilities library for quality filtering. This library was first introduced in (Eren et al. 2013) and its source code is available from https://github.com/meren/illumina-utils. The library contains the program merge-illumina-pairs that merges partially or fully overlapping paired-end Illumina reads and simultaneously performs quality filtering during the merging operation. To predict quality, it relies on the compliance between reads at the overlapped region instead of machine reported Q-scores, and eliminates reads based on the number of mismatches found at the overlapped region. When there is a mismatch in the overlapped region, the base to be used in the final merged sequence is picked from the read that possesses the higher Q-score base. However, if there is a disagreement between two reads, and neither of the reads have a Q-score higher than a minimum acceptable Q-score (which is Q10 by default), the corresponding position is denoted with an N in the merged read. Although the quality filtering with merge-illumina-pairs is done based only on the mismatches found in the overlapped region by default, it also offers a flag, --enforce-Q30-check, to enforce a final control on the parts of merged reads that did not overlap. This flag turns on the Q30 check,

which was originally explained by Minoche et al. (Minoche et al. 2011). Briefly, Q30-check assumes low-quality if the 66% of bases in the first half of a given read do not have Q-scores over Q30. merge-illumina-reads apply Q30 check only to the parts of reads that did not overlap, and discards the merged sequence if either of reads fail Q30 check. We merged and quality filtered the sponge microbiome dataset allowing up to three mismatches at the ~125 nt overlapped region, and using --enforce-Q30-check flag. Our quality-filtering steps removed 1,851,237 of 3,283,017 (56%) total number of reads from the sponge datasets. We found a large number of nonspecific 18S rRNA reads in our sponge samples best matching to *Hexadella* spp. in NCBI's nr database. Removal of these reads resulted in relatively low recovery of reads from the sponge samples compared to others. The average number of reads per sample retained following the quality filtering was 59,657 (min: 5,532; max: 208,213; std: 51,379) for the sponge datasets (See Supplementary Table S2 for details).

References

Eren AM, Vineis JH, Morrison HG, Sogin ML. (2013). A Filtering Method to Generate High Quality Short Reads Using Illumina Paired-End Technology Jordan, IK, ed. PLoS One 8:e66643.

Minoche AE, Dohm JC, Himmelbauer H. (2011). Evaluation of genomic high-throughput sequencing data generated on Illumina HiSeq and genome analyzer systems. Genome Biol. 12:R112.
