# Supplementary material for: Minimum entropy decomposition: Unsupervised oligotyping for sensitive partitioning of high-throughput marker gene sequences
Source: ISME J. 2014 Oct 17;9(4):968–79. doi: 10.1038/ismej.2014.195 (PMC4817710; doi:10.1038/ismej.2014.195)
Supplement: Supplementary Table 1 [file ismej2014195x4.doc]

| **Primer** | **Sequence** | **Type** |
| --- | --- | --- |
| Illumina R1 | AATGATACGGCGACCACCGAGATC- TACACTCTTTCCCTACACGACGCT- CTTCCGATCT | Bridge/ Sequencing adapter |
| Illumina R2 | CAAGCAGAAGACGGCATACGAGAT-xxxxxxGTGACTGGAGTTCAGACG- TGTGCTCTTCCGATCT | Bridge / Sequencing adapter, 6nt index |
| 518F | CCAGCAGCYGCGGTAAN | v4-v5 forward primer |
| 926R1 | CCGTCAATTCNTTTRAGT | v4-v5 reverse primer |
| 926R3 | CCGTCAATTTCTTTGAGT | v4-v5 reverse primer |
| 926R4 | CCGTCTATTCCTTTGANT | v4-v5 reverse primer |
