# Supplementary material for: Minimum entropy decomposition: Unsupervised oligotyping for sensitive partitioning of high-throughput marker gene sequences
Source: ISME J. 2014 Oct 17;9(4):968–79. doi: 10.1038/ismej.2014.195 (PMC4817710; doi:10.1038/ismej.2014.195)
Supplement: Supplementary Figure and Table Legends [file ismej2014195x7.doc]

# Supplementary Information

Supplementary Figure S1. Runtime performance of MED raw decomposition process compared to UCLUST.

Supplementary Figure S2. Alluvial diagram of the relationship between the top 100 MED nodes, OTUs and taxa in the oral microbiome dataset. The bottom panel expands the distribution of OTUs and MED nodes exemplified in Figure 5 with *Streptococcus, Fusobacterium, Neisseria,* and *Bacteroides.*

Supplementary Table S1. 16S-specific primers and the sequencing adaptors for paired-end sequencing on the Illumina MiSeq platform using 2 x 250 cycles.

Supplementary Table S2. Number of reads per sample, counts matrices and representative sequences for taxa, 3% OTUs and MED nodes for the sponge dataset.

Supplementary Table S3. Number of reads per sample, counts matrices and representative sequences for taxa, 3% OTUs, oligotypes, and MED nodes for the oral microbiome dataset.

Supplementary Methods. Supplementary methods.
